# Risk of subsequent self-harm, suicide attempts and suicide following a first hospital-treated self-harm episode among young people: a population-based cohort study

**DOI:** 10.1136/bmjment-2026-302569

**Published:** 2026-06-25

**Authors:** Thuy-Dung Nguyen, Moa Karemyr, Ralf Kuja-Halkola, Brian M D’Onofrio, Zheng Chang, Isabell Brikell, Paul Lichtenstein, Henrik Larsson, Patrick Sullivan, Yi Lu, Johan Bjureberg

**Affiliations:** 1 Department of Medical Epidemiology and Biostatistics, Karolinska Institutet, Stockholm, Sweden; 2 Department of Clinical Neuroscience, Karolinska Institutet, Stockholm, Sweden; 3 Department of Psychological and Brain Sciences, Indiana University Bloomington, Bloomington, Indiana, USA; 4 Department of Biomedicine, Aarhus University, Aarhus, Denmark; 5 School of Medical Sciences, Örebro University, Örebro, Sweden; 6 UNC Center for Psychiatric Genomics, University of North Carolina at Chapel Hill, Chapel Hill, North Carolina, USA

**Keywords:** Adolescent, Mental Health

## Abstract

**Background:**

Self-harm in young people is associated with elevated risks for subsequent self-harm, suicide attempts and suicide, particularly during the first year. Yet the trajectory across sex, age and self-harm methods remains poorly understood.

**Objective:**

To estimate risk for subsequent self-harm, suicide attempts and suicide following a first hospital-treated self-harm event in young people.

**Methods:**

This study included 77 647 individuals (57.0% female) whose first hospital-treated (ie, within inpatient or outpatient specialised healthcare) self-harm episode occurred between ages 10–24 years during 1973–2019. We estimated cumulative incidence and incidence rate for subsequent self-harm, suicide attempts and suicide at 1 month, 3 months and 1 year following the initial episode.

**Findings:**

Within 1 year, the cumulative incidence was 17.3% (95% CI 17.0 to17.5) for subsequent self-harm, 8.3% (8.1 to 8.5) for suicide attempt and 0.3% (0.2 to 0.3) for suicide. The highest risks occurred in the first month: 8.4% (8.2 to 8.6) for self-harm, 2.9% (2.8 to 3.0) for suicide attempt and 0.04% (0.03 to 0.05) for suicide. In the first month, the incidence rate of self-harm was 2.97 per 1000 person-days (2.90 to 3.05), falling to 0.55 (0.54 to 0.56) over the year. The suicide attempt rate declined from 0.98 (0.94 to 1.02) to 0.24 (0.24 to 0.25) and the suicide rate from 0.013 (0.009 to 0.019) to 0.007 (0.006 to 0.008). Males exhibited the highest suicide risk and females the highest attempts risk. First-month self-harm risk was greatest among males and children aged 10–12.

**Conclusions:**

The month following a self-harm episode is marked by an elevated risk of subsequent self-harm, suicide attempts and suicide, yet risk remains elevated over the full year.

**Clinical implications:**

These findings underscore the need for both acute and sustained prevention efforts. Special attention should be given to males and children aged 10–12 presenting with self-harm of ambiguous intent, as their risk of repetition may otherwise go unrecognised.

**WHAT IS ALREADY KNOWN ON THIS TOPIC**

- Self-harm is common among young people and is a major risk factor for suicide, repeated self-harm and subsequent suicide attempts. The year following a first self-harm episode represents a period of heightened risk for repetition.

**WHAT THIS STUDY ADDS**

- This population-based study demonstrates that for young people, the risk of subsequent self-harm, suicide attempt and suicide is particularly high during the first month following the initial self-harm episode that was treated within inpatient or outpatient specialised healthcare, referred to as hospital-treated. Furthermore, we show that the absolute risks of subsequent self-harm are particularly high among males and children aged 10-12 years and that using violent or multiple methods in that first recorded self-harm event is associated with increased risk of all outcomes.

**HOW THIS STUDY MIGHT AFFECT RESEARCH, PRACTICE OR POLICY**

- The period immediately following a first hospital-treated self-harm episode represents a critical window of heightened vulnerability in young people. These findings have implications for clinical risk assessment and post-self-harm care planning particularly with respect to early, developmentally appropriate and sex-sensitive prevention strategies.

## Background

 Suicide is a leading cause of death among young people aged 10–24 years.^1^ Worldwide annual suicide rates range between 3.0 and 15.5 per 100 000 for young males and 0.9 and 10.3 per 100 000 for young females.^1^ Self-harm is a major risk factor for suicide.^2^ One in five adolescents will engage in self-harm at some point.^3^ While some engage in self-harm only once, many remain at risk for subsequent self-harm episodes, suicide attempts and suicide.^4^,^6^ Identifying the incidence of subsequent episodes among young people, as well as the time frame in which the majority of episodes occur, is essential to guide risk assessment, resource allocation and treatment planning.

Longitudinal studies consistently demonstrate a markedly elevated risk of self-harm repetition and suicide during the first year following an index self-harm episode.^7^,^9^ Across cohorts from North America, Ireland and Australia, approximately 17%–20% of young people repeat self-harm within 1 year, and suicide incidence rates range around 3 per 1000 person-years.^4 7 10^ Suicide risk during this period has been estimated to be up to 30 times higher than in the general population.^9^ However, data on suicide attempts following a first self-harm event remain limited, and risk variation by method, intent, sex and age is not well understood.

Violent self-harm methods (eg, firearm use, jumping from heights) are associated with greater suicidal intent and risk than less violent methods (eg, cutting, burning)^11 12^ and are used more commonly by males.^12^ However, less violent methods requiring inpatient care are also associated with elevated suicide risk.^12^ Additionally, while young females attempt suicide more often than young males,^13^ males die by suicide at higher rates.^14^ Whether this same ‘gender paradox’ holds following an initial self-harm episode is less clear. Previous findings on sex differences in repetition, suicide attempts and suicide among young people appear inconsistent.^6 7 9 10 13^ These inconsistencies might be due to sample characteristics (eg, age span, geographical location) but are also likely related to differences in study design, specifically, in how self-harm, suicide attempts and suicide were defined, and whether self-harm of undetermined intent was incorporated into these definitions, in accordance with recommendations in suicide research.^15^ Thus, further research is needed to clarify sex-specific risks.

## Objective

Given evidence that the year following a first self-harm episode treated within inpatient or outpatient specialised healthcare, from here on referred to as hospital-treated episode, is a period of heightened risk, high-resolution longitudinal studies are needed to characterise outcomes immediately after a first episode and across the first year in young people. The primary objective of this study was to examine risks of subsequent self-harm, suicide attempts and suicide among young people, with particular attention to the first month, first 3 months and first year following the first hospital-treated episode. Additionally, the secondary objective was to assess differences in risk by sex, age and method of initial self-harm.

## Methods

We estimated the absolute risks of subsequent self-harm, suicide attempts and suicide following the first hospital-treated self-harm episode (ie, index self-harm episode) using cumulative incidence and calculated incidence rates of these outcomes during follow-up. Absolute risk was assessed continuously and at 1 month (30 days), 3 months (90 days) and 1 year (365 days). We stratified estimates by sex, age (10–12 years, 13–17 years, 18–24 years) and index method.

### Study design and population

We included individuals born between 1 January 1973 and 31 December 1996, allowing identification of index self-harm episodes occurring between ages 10 and 24 within the available register data (1 January 1973 to 31 December 2020). Individuals were eligible for follow-up if their first hospital-treated self-harm episode occurred during this age range and period. Participants were followed from the date of discharge from the index self-harm episode to identify outcomes, including subsequent self-harm, suicide attempts or suicide. For clinical relevance, the primary analyses focused on outcomes occurring within the first year following discharge. Sex-specific cumulative incidence analyses were additionally extended to 5 years to capture longer-term risk patterns.

### Register data

This study used data from the Swedish National Patient Register and the Cause of Death Register. Individual records were linked across registers and to the Total Population register—which includes information on migration status—using Swedish personal identity numbers assigned at birth or on immigration. The National Patient Register includes psychiatric inpatient records since 1973 and outpatient records from specialists since 2001, with near-complete coverage for inpatient care and approximately 80% coverage for outpatient care. Hospital-treated episodes of self-harm and suicide attempt were recorded using International Classification of Diseases (ICD) codes. The Cause of Death Register contains information on all deaths in Sweden since 1952, with the underlying cause of death recorded using ICD codes. Given the nature of the data, no variable contained missing values. For more information on the registers, see table S1 in the online supplemental file 1.

### Phenotype definitions

Non-fatal self-harm was identified in the National Patient Register using ICD codes for intentional self-harm (ICD-10 codes X60-X84 and ICD-8/9 codes E950-E958) and self-harm events of undetermined intent (ICD-10 codes Y10-Y34 and ICD-8/9 codes E980-E988).

Non-fatal suicide attempt was defined in the National Patient Register as any record with ICD codes for self-harm involving violent methods (firearm, jumping from heights, motor vehicle crash, suffocation or poisoning by gas) and/or requiring hospitalisation. Using ICD codes to distinguish suicide attempts from self-harm without suicidal intent is challenging due to the lack of information on intent. Combining information on self-harm methods with the level of healthcare contact (eg, inpatient care) provides an indicator of severity and suicide risk,^12^ which was used to distinguish suicide attempts, in line with previous research.^12 13^

Suicide was identified in the Cause of Death Register using ICD codes for any self-harm (ICD-10 codes X60-X84, Y10-Y34 and ICD 8/9 codes E950-E958, E980-E988).

ICD codes of events with undetermined intent were included to improve sensitivity and avoid underestimation of frequency of events, according to recommendations for suicide research.^15^

Defining subsequent self-harm using register records is challenging, with no established methods in prior studies. For transparency, we defined episodes pragmatically by grouping overlapping admission/discharge dates into one episode (see supplementary methods S3 in the online supplemental file 1 for other approaches that we explored).

For all index and subsequent self-harm episodes and suicide attempts, we only considered non-fatal cases, that is, not including those who died within the context of the episode—defined as having any record of being discharged with deceased status or date of death by self-harm during the defined episode.

To investigate risk by subgroups, we extracted information on sex, calculated age at index self-harm and defined methods of initial self-harm based on ICD codes (detailed definitions with ICD codes in table S1 in the online supplemental file 1).

### Statistical analyses

For each outcome, including subsequent self-harm, suicide attempts and suicide, we estimated the overall survival function S(t) across calendar days since the index self-harm using the Kaplan–Meier method and calculated the cumulative incidence as 1 − S(t). We then estimated covariate-adjusted cumulative incidence by standardising over covariate groups (sex, age at first self-harm and method of self-harm) using a Cox proportional hazards model. Finally, we estimated subgroup-specific cumulative incidence using Kaplan–Meier methods. If the outcome did not occur, individuals were censored at the earliest of the following: the end of 1-year or 5-year follow-up after the index self-harm episode, emigration, death from any cause (for self-harm and suicide attempt outcomes) or death from causes other than suicide (for the suicide outcome). Additionally, we estimated the incidence rate of subsequent self-harm, suicide attempts and suicide per 1000 person-days among individuals with an index self-harm (methods S1, S2 in the online supplemental file 1). Data management and analyses were conducted using R V.4.5.1 (software details in the online supplemental file 1).

For sensitivity analyses, we (1) estimated cumulative incidence of suicide death considering non-suicide death as competing using the Aalen-Johansen estimator and (2) compared the risk of outcomes between three groups: (a) individuals with index self-harm of determined intent (ICD-10X60–X84; ICD-8/9 E950–E958), (b) individuals with index self-harm of undetermined intent (ICD-10 Y10–Y34; ICD-8/9 E980–E988) and (c) individuals without self-harm using a matched cohort study (methods S3 in the online supplemental file 1).

## Findings

### Population characteristics

We defined a base population of 3 646 589 individuals who were born between 1973 and 1996, alive and did not emigrate before the age of 10. Among the cohort, 78 025 individuals had an initial self-harm episode at ages 10–24 during the follow-up period 1973–2019. Of these, 366 (0.47%) died by suicide and 12 (0.02%) from other causes during the treatment period of the initial episode, resulting in 77 647 individuals included in analyses.

The analysed population (ie, index population) included 77 647 individuals, of which 44 238 (56.97%) were females. The mean age at first self-harm was 19.08 years (SD=3.43). Most individuals were aged 18 to 24 years (70.52%), and poisoning was the most common method used in the index self-harm episode (56.96%) (table 1).

**Table 1:** Characteristics of index population

Characteristic		Total population (n=77 647)	Female (n=44 238; 56.97%)	Male (n=33 409; 43.03%)
Age at index self-harm		Range: 10.00–24.99 First-third quartiles: 16.48–21.89 Median: 19.21 Mean: 19.08 SD: 3.43	Range: 10.00–24.99 First-third quartiles: 16.11–21.40 Median: 18.67 Mean: 18.73 SD: 3.33	Range: 10.00–24.99 First-third quartiles: 17.15–22.40 Median: 19.95 Mean: 19.55 SD: 3.50
Age group at index self-harm	10-12 years	1626 (2.09%)	740 (1.67%)	886 (2.65%)
	13-17 years	21 262 (27.38%)	14 146 (31.98%)	7116 (21.30%)
	18-24 years	54 759 (70.52%)	29 352 (66.35%)	25 407 (76.05%)
Method used in index self-harm*	Poisoning	44 228 (56.96%)	30 373 (68.66%)	13 855 (41.47%)
	Tissue damage	9295 (11.97%)	4911 (11.10%)	4384 (13.12%)
	Violent	3114 (4.01%)	1141 (2.58%)	1973 (5.91%)
	Multiple	489 (0.63%)	301 (0.68%)	188 (0.56%)
	Unspecified	20 521 (26.43%)	7512 (16.98%)	13 009 (38.94%)

* Registered diagnostic codes according to the International Classification of Diseases (ICD-8/9, ICD-10), divided into the following categories: poisoning (ICD-10: X60-X66, X68, X69, Y10-Y16, Y18, Y19, ICD-8/9: E950, E980); tissue damage (ICD-10: X76-X79, Y26-Y29, ICD-8/9: E956, E986); violent methods (ICD-10: X67, X70-X75, X80-X82, Y17, Y20-Y25, Y30-Y32, ICD-8/9: E951-E955, E957, E981-E985, E987); and unspecified (ICD-10: X83, X84, Y33, Y34, ICD-8/9: E958, E988).

### Incidence of subsequent self-harm, suicide attempts and suicide

The cumulative incidence estimates indicate that within 1 year of an index self-harm episode, 17.25% (95% CI 16.98 to 17.52) had a subsequent self-harm event, 8.33% (95% CI 8.14 to 8.53) attempted suicide and 0.26% (95% CI 0.23 to 0.30) died by suicide (table 2). After standardising over covariates (sex, age at first self-harm and method of self-harm), the estimated risks were slightly lower for non-fatal outcomes: 13.10% (95% CI 12.67 to 13.53) for subsequent self-harm and 8.20% (95% CI 7.80 to 8.60) for suicide attempt. In contrast, the incidence for suicide was higher at 0.36% (95% CI 0.27 to 0.45). The highest incidence for repeat self-harm, suicide attempt and suicide were observed within the first month following the initial episode (figure S1 in the online supplemental file 1).

**Table 2:** Total cumulative incidence and incidence rate

Outcome		N individuals with outcome	Cumulative incidence (%)		Incidence rate (per 1000 person-days)		
			Crude (95% CI)	Adjusted* For covariates (95% CI)	Total thousand person-day	N event	Crude rate (95% CI)†
Subsequent self-harm	1 month	6527	8.41 (8.21 to 8.60)	6.29 (6.05 to 6.53)	2196.18	6527	2.9720 (2.9003 to 3.0450)
	3 months	8882	11.44 (11.22 to 11.67)	8.61 (8.30 to 8.92)	6376.86	8882	1.3928 (1.3640 to 1.4221)
	1 year	13 371	17.25 (16.98 to 17.52)	13.10 (12.67 to 13.53)	24 490.39	13 371	0.5460 (0.5368 to 0.5553)
Suicide attempt	1 month	2236	2.88 (2.76 to 3.00)	2.80 (2.63 to 2.97)	2282.39	2236	0.9797 (0.9395 to 1.0211)
	3 months	3606	4.65 (4.50 to 4.79)	4.53 (4.29 to 4.78)	6755.42	3606	0.5338 (0.5165 to 0.5515)
	1 year	6455	8.33 (8.14 to 8.53)	8.20 (7.80 to 8.60)	26 572.92	6455	0.2429 (0.2370 to 0.2489)
Suicide	1 month	31	0.04 (0.03 to 0.05)	0.05 (0.03 to 0.08)	2328.52	31	0.0133 (0.0090 to 0.0189)
	3 months	74	0.10 (0.07 to 0.12)	0.13 (0.09 to 0.17)	6981.24	74	0.0106 (0.0083 to 0.0133)
	1 year	205	0.26 (0.23 to 0.30)	0.36 (0.27 to 0.45)	28 233.90	205	0.0073 (0.0063 to 0.0083)

* Adjusted for sex, age at index self-harm (continuous) and method used in index self-harm.

† Four decimal places are shown to capture small variations in suicide outcome estimates.

The rates of subsequent self-harm and suicide attempts were highest within the first 15 days and declined sharply thereafter, whereas trends in suicide rates were less clearly defined (figure S1 in the online supplemental file 1). In the first month, the crude incidence rate for subsequent self-harm was 2.97 per 1000 person-days (95% CI 2.90 to 3.05), compared with 0.55 (95% CI 0.54 to 0.56) over the full year. The incidence rate of suicide attempts declined from 0.98 (95% CI 0.94 to 1.02) to 0.24 (95% CI 0.24 to 0.25) per 1000 person-days, while the suicide rates decreased from 0.0133 (95% CI 0.0090 to 0.0189) to 0.0073 (95% CI 0.0063 to 0.0083) over the same period (table 2).

Considering all-cause death as a competing event for subsequent self-harm and suicide attempt, and non-suicide death for suicide death, sensitivity analyses showed cumulative incidence estimates consistent with the primary results (figure S3 in the online supplemental file 1).

### Incidence by subgroups

#### Sex-specific incidence

**Figure 1:**
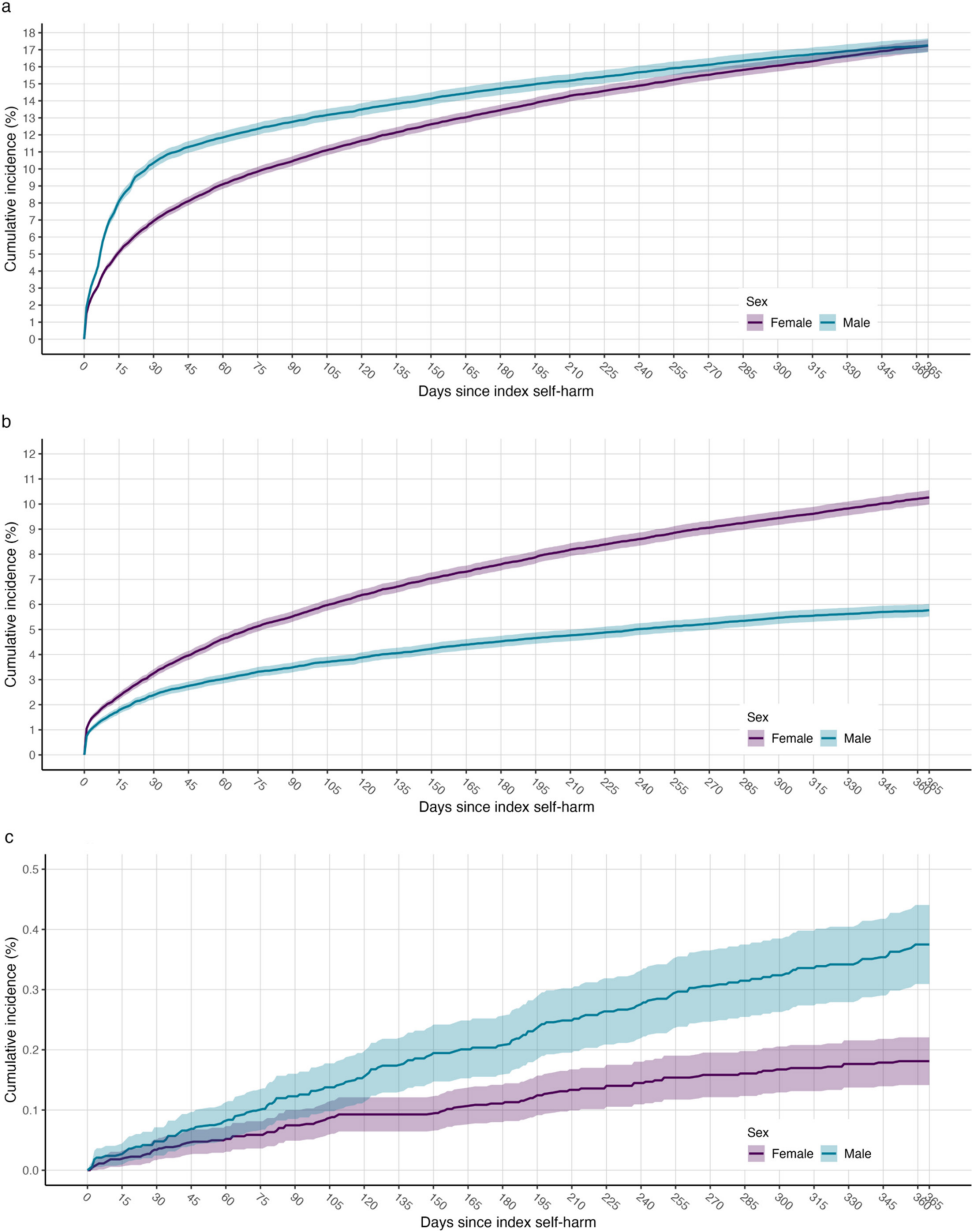
Cumulative incidence *by sex* over calendar day since the index self-harm Incidence of (a) subsequent self-harm, (b) suicide attempt and (c) suicide cumulative incidence was estimated from crude models, not adjusted for any covariate, using the Kaplan-Meier method. Colours represent data separately by sexes. The lines show the proportion (%) of individuals who experienced an outcome among those still at risk on each corresponding day shown on the x-axis. Shading regions show 95% CIs of the estimates.

Within the first year after the index self-harm, 17% of both sexes repeated self-harm. Males showed a rapid increase in incidence during the first 30 days, which then levelled off, while females experienced a steadier increase throughout the year (figure 1). After 1 year, the incidence was consistently higher in females (figure S4 in the online supplemental file 1).

Suicide attempts occurred in 10% of females and 6% of males during the first year. Females consistently had a higher incidence of suicide attempts across the follow-up period—both within the first year and beyond—across all age groups except ages 10–12 (figure 1, figures S4, S5 in the online supplemental file 1).

Although suicide remained rare (<0.5%) in both groups during the first year, the proportion was more than twice as high in males (n=125; 0.37%) compared with females (n=80; 0.18%) (table S2 in the online supplemental file 1). The incidence of suicide remained higher among males during the first year and up to 5 years after the index event (figure 1, figure S4 in the online supplemental file 1).

#### Age-specific incidence

**Figure 2:**
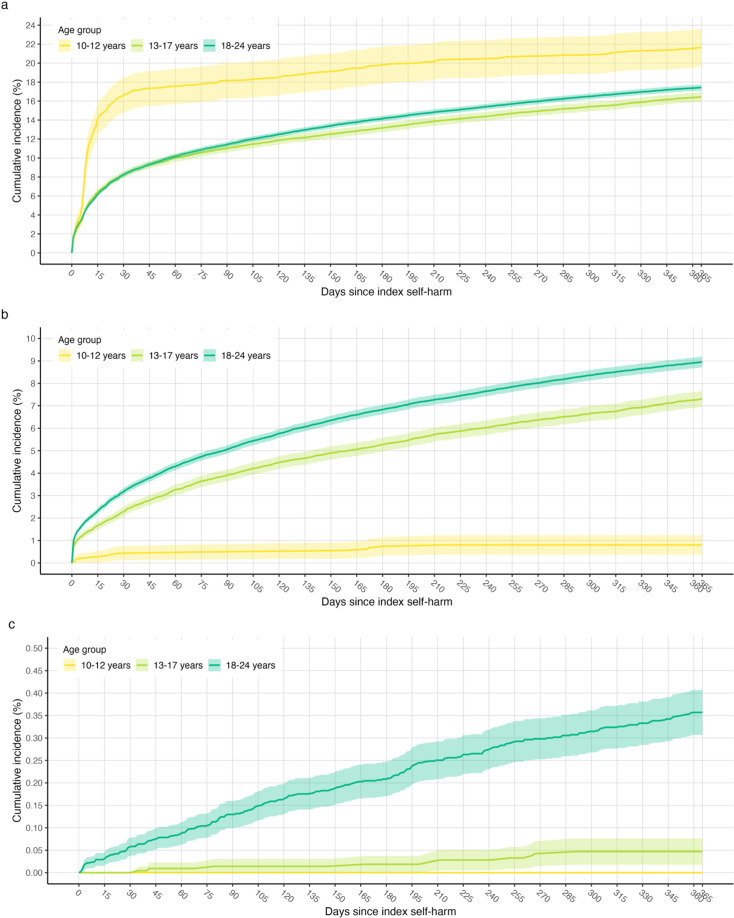
Cumulative incidence *by age group* over calendar day since the index self-harm Incidence of (a) subsequent self-harm, (b) suicide attempt and (c) suicide cumulative incidence was estimated from crude models not adjusted for any covariate, using the Kaplan-Meier method. Colours represent data separately by age groups. The lines show the proportion (%) of individuals with an outcome among individuals who were still at risk at corresponding day on the x-axis. Shading regions show 95% CIs of the estimates.

Within the first year after index self-harm, the cumulative incidence of repeated self-harm was 22% in ages 10–12, 16% in ages 13–17 and 17% in ages 18–24. For suicide attempts, cumulative incidence was under 1% for the youngest group and 7%–9% for the older groups. Suicide was rare across all ages, with no cases among children under 12 (figure 2, table S2 in the online supplemental file 1).

#### Incidence specific for methods used in first self-harm

Although comprising less than 5% of the index population, individuals who used violent or multiple methods had the highest cumulative incidence of repeated self-harm and suicide attempt: 21% (violent methods) and 28% (multiple methods) repeated self-harm, while 19% and 18% attempted suicide within the first year, respectively. In comparison, those who used poisoning or tissue damage had lower cumulative incidence—repeated self-harm occurred in 14% and 20% and suicide attempts in 11% and 7%, respectively (figure S2, table S2 in the online supplemental file 1). Notably, individuals with unspecified methods had the highest cumulative incidence of repeated self-harm within the first 30 days (figure S2a in the online supplemental file 1). In terms of suicide, the group who used violent methods had the highest incidence across the first year (figure S2c in the online supplemental file 1).

## Discussion

This study examined the risk of subsequent self-harm, suicide attempts and suicide at three critical time points within the first year following a first hospital-treated self-harm episode in young people. Within the first year, cumulative incidences were 17.25% for repeated self-harm, 8.33% for suicide attempt and 0.26% for suicide. Notably, the incidence rates were highest during the first month. Males exhibited the highest suicide risk, whereas females showed a greater risk for suicide attempts. Although males and children aged 10–12 years had lowest risk for subsequent suicide attempt over the full year, during the first month, they had the highest risk for subsequent self-harm when episodes of undetermined intent were included. Violent and multiple methods of self-harm were associated with the greatest risks of both repeated self-harm and suicide attempt.

The cumulative incidence of repeated self-harm within the first year was comparable to previous findings in young people.^4 7 10^ The particularly high incidence rates of all outcomes observed during the first month are in line with results from a Swedish cohort study including individuals of all ages,^13^ as well as findings in adults.^16 17^ Given the peak of self-harm in adolescence,^18^ the actual proportion of young people experiencing non-fatal and fatal outcomes following an initial self-harm episode is notably high. These findings suggest that the period immediately following a first self-harm episode may represent a particularly critical window of vulnerability, with implications extending beyond the first month.

Sex differences were observed. The rate of suicide was more than twice as high in males (n=125; 0.37%) as in females (n=80; 0.18%). This aligns with previous findings for young people,^7 9^ but contrasts with a recent Swedish study that reported no sex differences in suicide.^19^ The substantially larger statistical power of the present study may explain this discrepancy. During the first year, males—except those aged 10–12 years—had higher rates of repeated self-harm, whereas females exhibited higher rates thereafter, a pattern also reported in other large health record–based studies.^7 10^ Consistent with findings in adults,^20^ females had a higher cumulative risk of suicide attempt. In contrast, a previous population-based study including individuals of all ages reported a different sex pattern,^13^ which may reflect a broader outcome definition.

Violent and multiple methods of self-harm were associated with the highest risks of repeated self-harm and suicide attempt. This aligns with findings of higher suicidal intent^11^ and increased risk of both repeated self-harm^21^ and suicide^4 11 12^ in association with violent and multiple methods. Together, these findings underscore the need for targeted interventions and close clinical monitoring of individuals engaging in such behaviours. Healthcare should provide means restriction counselling at discharge to these young people as well as their caregivers. During the first month, however, the highest risk for repeated self-harm was observed for those who self-harmed using unspecified methods (including events of both determined and undetermined intent where the method was not specified). This was also the second most common index method across sexes. We hypothesise that clinical management may play a role: since recommended next care following self-harm has been shown to vary by method, with more lethal methods being associated with more intensive follow-up,^22^ presentations with unspecified methods may receive lower-intensity post-discharge monitoring, potentially leaving imminent risk undetected. Additionally, individuals presenting with unspecified methods may represent a clinically complex group, where the absence of a clearly identifiable method reflects the severity or disorganisation of the crisis itself rather than its absence. Taken together, these findings stress the importance of method-agnostic risk assessment that does not rely on a clearly identifiable method to trigger close post-discharge monitoring.

While self-harm of undetermined intent was included to mitigate underestimation of initial and subsequent self-harm as well as suicide attempt and suicide,^23 24^ it is possible that some of these events were not intentional. Nevertheless, although initial self-harm with determined intent was linked to a higher overall risk than initial self-harm with undetermined intent, we found that young people presenting with self-harm with undetermined intent had a substantially higher risk of subsequent self-harm, suicide attempt and suicide with determined intent compared with those with no history of self-harm (tables S3, S4 in the online supplemental file 1). This finding supports our definition, which includes both determined and undetermined intent, and underscores the importance of addressing self-harm presentations of undetermined intent. Further, young males in treatment for self-harm tend to report less suicidality and psychopathology, as well as greater quality of life,^25^ which likely would influence assessments of self-harm and suicide attempts. Indeed, it has been found that healthcare providers are more likely to classify self-harm in males as having undetermined intent^26^ as well as that self-harm events in males are more often under-reported.^27^ These findings further highlight the importance of considering potential sex-related biases in the classification of self-harm. Misclassification may obscure true incidence patterns and impact the accuracy of risk assessment and prevention efforts. This pattern may also contribute to the disproportionate representation of males among unspecified-method presentations observed in the present study (38.94% of males vs 16.98% of females), and may help explain why this group faces elevated short-term repetition risk: if ambiguous presentations in males are systematically under-assessed, imminent risk may go undetected at the critical post-discharge window. Such biases may also contribute to disparities in treatment research.^28^

Although self-harm, suicide attempt and suicide are less prevalent in preadolescent children, recent findings suggest that this population also engage in these behaviours.^29^ In this study, children aged 10–12 had the highest risk of repeated self-harm, despite experiencing fewer overall events. However, when the analyses were restricted to events of determined intent, this was reversed–the youngest group showed the lowest incidence of repeated self-harm. As discussed above, this might reflect a genuine difference in intent, or alternatively, a tendency to interpret self-harm in preadolescent children as of undetermined intent. These findings underscore the importance of assessing self-harm and suicide risk in younger children, especially following a self-inflicted injury regardless of apparent intent, as their suicidal risk may otherwise go unrecognised in clinical and research settings.

### Strengths and weaknesses

This retrospective cohort study’s strengths included a large sample size and data from population-based registers. The study also had limitations. Register-based data on self-harm outcomes using ICD codes lack information on suicidal intent. Assumptions about suicidal intent were based on the severity of the method and the need for hospitalisation.^12 13^ Given that individuals who self-harm severely without suicidal intent may be hospitalised, whereas suicide attempts using nonviolent methods may not lead to hospitalisation, it is possible that these assumptions created misclassification bias through overestimation or underestimation of true suicide attempts. Additionally, individuals who self-harm repetitively may underestimate the lethality of their method,^30^ potentially resulting in unintended need for inpatient care and further complicating the suicide attempt definition. Thus, future studies should validate this procedure to distinguish suicide attempts. Further, the self-harm cases included in this study likely represented more severe incidents and the rates of self-harm might be underestimated. Moreover, defining self-harm and suicide attempts using non-overlapping admission and discharge dates has its weaknesses, as it could lead to overestimation of subsequent events among individuals with an initial self-harm episode. However, given that the increased risk of outcomes persists beyond the first day and the first week, applying stricter definitions is unlikely to change the study’s overall conclusions. The risk of suicide was also highest the first days after the initial attempt and for every suicide there is likely several suicide attempts.^4 6 9^ Given this, a longer grace period might instead underestimate the risk of subsequent self-harm and suicide attempts. Further research is needed to identify an optimal method for defining subsequent self-harm or suicide attempts using registry data. In addition, the analyses did not account for potentially important confounders, including psychiatric diagnoses, socioeconomic factors and prior treatment history, all of which may influence self-harm trajectories and subsequent outcomes. These analyses were designed to reflect information typically available during routine clinical assessment, providing absolute risk figures that reflect the population-level burden of outcomes following a first hospital-treated self-harm episode and remain interpretable without requiring knowledge of individual psychiatric or socioeconomic profiles. Nevertheless, future studies should examine how these factors modify risk trajectories, particularly to identify subgroups where targeted interventions may be most beneficial. Finally, similar studies in other countries or regions are needed to assess the generalisability of the current findings.

## Clinical implications

This study demonstrated that the risks of repeated self-harm, suicide attempts and suicide death among young people with self-harm are particularly high during the first month following an initial hospital-treated self-harm episode, underscoring the importance of intensive follow-up care in this immediate post-discharge window. Discharge planning should not be deferred until repeat presentation: proactive outreach, structured safety planning and means restriction counselling should be initiated at the point of first contact. Given that risk is highest in the days immediately following discharge, follow-up contact within days rather than weeks is warranted, particularly for high-risk subgroups. Special attention should be directed towards males, who face elevated suicide risk and are disproportionately represented among unspecified-method presentations, a group that may be systematically under-assessed at triage. Clinicians should be aware that the absence of a clearly identifiable method does not indicate lower risk. Similarly, children aged 10–12 may require age-appropriate risk assessment that does not discount self-harm as unintentional based on young age alone. In contrast, for individuals engaging in violent or multiple methods, the substantially elevated risks of repeated self-harm and suicide attempt indicate a need for targeted intervention and close clinical monitoring. Among the treatments warranting consideration for young people in this high-risk period are those combining structured family involvement with emotion regulation skills training.^28^ However, given the concentration of risk in the immediate post-discharge period, brief interventions that can be delivered rapidly in acute care settings before longer-term therapy is initiated are particularly needed.

## Supplementary material

10.1136/bmjment-2026-302569 online supplemental file 1

## Data Availability

Data may be obtained from a third party and are not publicly available.
