# Supplementary material for: Risk of subsequent self-harm, suicide attempts and suicide following a first hospital-treated self-harm episode among young people: a population-based cohort study
Source: BMJ Ment Health. 2026 Jun 25;29(1):e302569. doi: 10.1136/bmjment-2026-302569 (PMC13311623; doi:10.1136/bmjment-2026-302569)
Supplement: online supplemental file 1 [file bmjment-29-1-s001.pdf]

Supplement for:

Risk of subsequent self-harm, suicide attempts, and suicide following a first hospital-treated self-harm episode among young people: A population-based cohort study

Authors:
Thuy-Dung Nguyen, Moa Karemyr, Ralf Kuja-Halkola, Brian D'Onofrio, Zheng Chang, Isabell Brikell, Paul Lichtenstein, Henrik Larsson, Patrick Sullivan, Yi Lu, Johan Bjureberg

Corresponding author:
Johan Bjureberg
Department of Clinical Neuroscience, Karolinska Institutet
Norra Stationsgatan 69, SE-131 64 Stockholm, Sweden
E-mail: johan.bjureberg@ki.se

Table of Contents

I. SUPPLEMENTARY METHODS 2

S1. Estimate cumulative incidence 2

S2. Estimate incidence rate 2

S3. Sensitivity analysis 2

S4. Statistical software and package versions 3

II. SUPPLEMENTARY TABLES 4

Table S1: Variable definition 4

Table S2: Number of outcomes by subgroups 5

Table S3: Characteristics and number of outcomes among individuals with index self-harm defined based on intention 6

Table S4: Hazard ratios of subsequent self-harm, suicide attempt and suicide within the year following initial self-harm, comparing individuals with index self-harm defined based on intention vs matched without a self-harm 7

III. SUPPLEMENTARY FIGURES 8

Figure S1: Incidence of outcomes across calendar day within 1 year since the index self-harm 8

Figure S2: Cumulative incidence by method of self-harm over calendar day since the index self-harm 9

Figure S3: Cumulative incidence of outcomes over calendar day since the index self-harm considering competing events 10

Figure S4: Cumulative incidence by sex over calendar day since the index self-harm during the first 5 years 11

Figure S5: Cumulative incidence by sex and age group over calendar day since the index self-harm 12

References 13

I. SUPPLEMENTARY METHODS

S1. Estimate cumulative incidence

The Kaplan–Meier estimator was used to estimate the survival function *S(t)* over the 1-year period following the first instance of self-harm, from which the crude cumulative incidence of self-harm, suicide attempt, and suicide was calculated as 1-*S(t)*. This analysis was conducted using the survfit() function from the survival package in R. Example of R code:

```
surv_fit_outcome <- survfit(Surv(start_time, end_time_outcome, outcome) ~ 1, data = my_data, timefix = FALSE, type="kaplan-meier")
```

To estimate the covariate-adjusted cumulative incidence, a Cox proportional hazards model was first fitted, adjusting for sex, age at first self-harm, and self-harm method. The predicted survivor function was then computed using survfit() based on this adjusted Cox model. Example of R code:

```
cox_fit_outcome <- survfit(coxph(Surv(start_time, end_time_outcome, outcome) ~ sex + age_first_self_harm + method_first_self_harm_group, data = my_data), type="kaplan-meier")
```

S2. Estimate incidence rate

The crude incidence rate was calculated as number of events divided by total person-day using the survRate() function from the biostat3 package. R code example for calculating rate per 1000 person-days:

```
IR_outcome <- survRate(Surv(start_time/1000, end_time_outcome /1000, outcome) ~ 1, data = my_data)
```

To visualize the incidence rate by day, we modeled the instantaneous failure rate—also referred to as the hazard function or risk function—using nonparametric smoothing, as implemented in the bshazard() function from the bshazard package. This method does not assume any underlying distribution for failure times. R code example:

```
Hazard_outcome = bshazard(Surv(start_time, end_time_outcome, outcome) ~ 1, data = my_data, degree = 1)
```

S3. Sensitivity analysis

To compare the risk of subsequent self-harm, suicide attempt, and suicide—each defined by events of determined intent—we conducted a matched cohort study including three groups: (1) individuals with index self-harm of determined intent (ICD-10 X60–X84; ICD-8/9 E950–E958), (2) individuals with index self-harm of undetermined intent (ICD-10 Y10–Y34; ICD-8/9 E980–E988), and (3) individuals without self-harm.

The base population included individuals born between 1973-01-01 and 1996-12-31, who were alive and had not emigrated before age 10 (as in the main analyses). From this population, we defined two exposed groups: those with index self-harm of determined or undetermined intent. For each exposed individual, we randomly selected five unexposed individuals—matched on sex and birth year—without any self-harm on the index date (the date of self-harm in the matched exposed individual). For exposed group 2 (undetermined intent), unexposed individuals also had no prior self-harm of determined intent by the index date.

Unexposed individuals were selected using incidence density sampling. We chose a 1:5 ratio as increasing the number of unexposed per case provides minimal additional precision.[1] Individuals could be selected as unexposed before potentially becoming exposed at a later time. We followed both exposed and unexposed groups for one year to observe three outcomes: subsequent self-harm, suicide attempt, and suicide.

We estimated incidence rates for each group and presented results stratified by sex and age group. To estimate hazard ratios, we used stratified Cox proportional hazards regression (via the coxph() function in R's survival package[2]), with days since the index date as the time scale. All models were stratified by matching cluster, thus inherently adjusting for sex and birth year.

For models of self-harm and suicide attempt, individuals were censored at the earliest of all-cause death, emigration, or 31/12/2020. For suicide models, individuals were censored at the earliest of death from other causes rather than suicide, emigration, or 31/12/2020.

There are no established methods to define subsequent self-harm using register records in prior studies. We explored various approaches, including analysing record characteristics within individuals (e.g., admission type, visit type, discharge department, emergency visits), but found no consistent patterns. We also tested time-based definitions using gaps of 1 to 365 days between records, assuming subsequent self-harm would resemble initial ones—again, no clear patterns emerged. We therefore combined episodes with overlapping admission and discharge dates to pragmatically define a single self-harm episode.

S4. Statistical software and package versions

Software and packages used for data analyesis, generated using sessionInfo()

R version 4.5.1 (2025-06-13 ucrt)
Platform: x86_64-w64-mingw32/x64
Running under: Windows 10 x64 (build 19045)

Matrix products: default
LAPACK version 3.12.1

locale:
LC_COLLATE=Swedish_Sweden.utf8

LC_CTYPE=Swedish_Sweden.utf8

LC_MONETARY=Swedish_Sweden.utf8
LC_NUMERIC=C

LC_TIME=Swedish_Sweden.utf8

time zone: Europe/Stockholm
tzcode source: internal

attached base packages:
splines stats graphics grDevices utils datasets methods base

other attached packages:
colorspace_2.1-0 treemapify_2.5.6 gridExtra_2.3 bshazard_1.1 Epi_2.47.1 survminer_0.4.9
ggpubr_0.6.0 biostat3_0.1.8 MASS_7.3-60 survival_3.5-5 DBI_1.1.3 lubridate_1.9.2
forcats_1.0.0 stringr_1.5.0 dplyr_1.1.2 purrr_1.0.1 readr_2.1.4 tidyr_1.3.0
tibble_3.2.1 ggplot2_3.4.4 tidyverse_2.0.0 data.table_1.14.8

loaded via a namespace (and not attached):
ggfittext_0.10.1 gtable_0.3.3 xfun_0.39 rstatix_0.7.2 etm_1.1.1 lattice_0.21-8
numDeriv_2016.8-1.1 tzdb_0.3.0 vctrs_0.6.2 tools_4.5.1 generics_0.1.3 parallel_4.5.1
fansi_1.0.4 pkgconfig_2.0.3 Matrix_1.5-4 lifecycle_1.0.3 compiler_4.5.1 munsell_0.5.0
carData_3.0-5 htmltools_0.5.5 yaml_2.3.7 pillar_1.9.0 car_3.1-2 muhaz_1.2.6.4
cmprsk_2.2-11 abind_1.4-5 nlme_3.1-162 km.ci_0.5-6 tidyselect_1.2.0 digest_0.6.31
stringi_1.7.12 fastmap_1.1.1 grid_4.5.1 cli_3.6.1 magrittr_2.0.3 utf8_1.2.3
broom_1.0.4 withr_2.5.0 scales_1.2.1 backports_1.4.1 timechange_0.2.0 rmarkdown_2.21
ggsignif_0.6.4 zoo_1.8-12 hms_1.1.3 evaluate_0.20 knitr_1.42 KMsurv_0.1-5
mgcv_1.8-42 survMisc_0.5.6 rlang_1.1.1 Rcpp_1.0.10 xtable_1.8-4 glue_1.6.2
rstudioapi_0.14 R6_2.5.1 plyr_1.8.8

II. SUPPLEMENTARY TABLES

Table S1: Variable definition

Variable	Definition	Cut-off/categories
Sex	Registered sex retrieved from the Total Population Register[3]25/06/2026 06:32:00	Binary (Male/Female)
Age at index self-harm	Age at the admission date of the first self-harm in year. Calculated from date of admission and date of birth.	Continuous variable and categorised into 3 groups:<br>1) 10-12 years<br>2) 13-17 years<br>3) 18-24 years
Methods used in self-harm/suicide attempt	Defined based on the 3 first characters of the ICD-10 codes, and the 4 first characters of the ICD-8/9 codes. If multiple codes are recorded for a self-harm or suicide attempt, select the code with the highest priority based on the following hierarchy: X code → E95 code → Y code → E98 code. If multiple codes share the same priority, select the one with the earliest admission date.	9 categories<br>1) Drug/poison: X60, X61, X62, X63, X64, X65, X66, X68, X69, Y10, Y11, Y12, Y13, Y14, Y15, Y16, Y18, Y19, E950, E980<br>2) Firearm: X72, X73, X74, X75, Y22, Y23, Y24, Y25, E955, E985<br>3) Gas: X67, Y17, E951, E952, E981, E982<br>4) Heat: X76, X77, Y26, Y27<br>5) Jump/crash: X80, X81, X82, Y30, Y31, Y32, E987, E957<br>6) Cutting: X78, Y28, E956, E986<br>7) Blunt object: X79, Y29<br>8) Suffocation: X70, X71, Y20, Y21, E953, E954, E983, E984<br>9) Others and unspecified: X83, X84, Y33, Y34, E958, E988
Method groups used in self-harm/suicide attempt	Grouping of the 'Methods used in self-harm/suicide attempt' (see above).	5 categories<br>1) Multiple: More than 1 method recorded within the treatment period<br>2) Poisoning: Drug/poison<br>3) Tissue damage: Cutting, blunt objects, heat<br>4) Violent: Firearm, gas, jump/crash, suffocation<br>5) Unspecified: Others and unspecified
Index self-harm	Defined from Patient Register as patient record with ICD-10 codes X60-X84, Y10-Y34, and ICD 8/9 codes E950-E958, E980-E988.<br>Extract the first episode; then exclude those who died within the context of the first episode (see definition below).	Binary variable (Yes/No)
Subsequent self-harm	Defined from Patient Register as patient record with ICD-10 codes X60-X84, Y10-Y34, and ICD 8/9 codes E950-E958, E980-E988.<br>Extract the 2nd episode, then remove individuals who died within the 2nd episode.	Time to event.<br>Defined from date of first record of first episode to date of first record of 2nd episode
Suicide attempt	Defined from Patient Register as Patient records with ICD-10 codes X60-X84, Y10-Y34 and ICD8/9 codes E950-E958, E980-E988, with:<br>a. A notably violent method of self-harm (firearm, jumping from heights, motor vehicle crash, suffocation, or poisoning by gas) and/or<br>b. Led to inpatient care[4]<br>We excluded those who died within the context of the suicide attempt.	Time to event.<br>Defined from date of first record of first episode to date of first record of the suicide attempt episode
Suicide	Defined using data from Cause of Death register as any death record with ICD-10 codes X60-X84, Y10-Y34, and ICD-8/9 codes E950-E958, E980-E988[5]	Time to event.<br>Defined from date of first record of first episode to date death due to suicide
Death within the context of a self-harm or suicide attempt episode	After concatenating all records within each episode, we defined death within the episode as the presence of any record indicating discharge with a deceased status or a date of death by self-harm occurring within the treatment period of that episode.	Binary variable (Yes/No)

Table S2: Number of outcomes by subgroups

Characteristic		Subsequent self-harm			Suicide attempt			Suicide		
		1 month N (%)	3 months N (%)	1 year N (%)	1 month N (%)	3 months N (%)	1 year N (%)	1 month N (%)	3 months N (%)	1 year N (%)
Sex	Female	3069 (6.94)	4621 (10.45)	7614 (17.21)	1440 (3.26)	2441 (5.52)	4532 (10.24)	15 (0.03)	33 (0.07)	80 (0.18)
	Male	3458 (10.35)	4261 (12.75)	5757 (17.23)	796 (2.38)	1165 (3.49)	1923 (5.76)	16 (0.05)	41 (0.12)	125 (0.37)
Age group at first self-harm	10-12 years	271 (16.67)	295 (18.14)	352 (21.65)	7 (0.43)	8 (0.49)	13 (0.80)	0 NA	0 NA	0 NA
	13-17 years	1752 (8.24)	2341 (11.01)	3494 (16.43)	486 (2.29)	831 (3.91)	1552 (7.30)	0 NA	<5 NA	10 (0.05)
	18-24 years	4504 (8.23)	6246 (11.41)	9525 (17.39)	1743 (3.18)	2767 (5.05)	4890 (8.93)	31 (0.06)	71 (0.13)	195 (0.36)
Method used in first self-harm	Poisoning	2011 (4.55)	3344 (7.56)	6329 (14.31)	1528 (3.45)	2555 (5.78)	4870 (11.01)	18 (0.04)	46 (0.10)	136 (0.31)
	Tissue damage	935 (10.06)	1295 (13.93)	1892 (20.36)	241 (2.59)	373 (4.01)	646 (6.95)	<5 NA	13 (0.14)	28 (0.30)
	Violent	355 (11.40)	501 (16.09)	651 (20.91)	345 (11.08)	480 (15.41)	602 (19.33)	8 (0.26)	12 (0.39)	22 (0.71)
	Multiple	49 (10.02)	84 (17.18)	139 (28.43)	25 (5.11)	50 (10.22)	87 (17.79)	<5 NA	<5 NA	<5 NA
	Unspecified	3177 (15.48)	3658 (17.83)	4360 (21.25)	97 (0.47)	148 (0.72)	250 (1.22)	<5 NA	<5 NA	16 (0.08)

Table S3: Characteristics and number of outcomes among individuals with index self-harm defined based on intention

We identified 48,300 individuals who had an index self-harm defined using International Statistical Classification of Diseases and Related Health Problems (ICD) codes for events of determined intent using ICD-10 codes X60-X84 and ICD-8/9 codes E950-E958; and 29,532 individuals who had an index self-harm defined using ICD codes for events of undetermined intent using ICD-10 codes Y10-Y34 and ICD 8/9 codes E980-E988) during age 10-24 during the follow-up.

**Outcome defined based on events of determined intent**		**Index self-harm defined based on events of *determined* intent; N (%)**		**Index self-harm defined based on events of *undetermined* intent; N (%)**	
		**With self-harm (N = 48,300)**	**Matched without self-harm (N = 241,500)**	**With self-harm (N = 29,532)**	**Matched without self-harm (N = 147,660)**
**Characteristics**					
Female sex (%)		32,798 (67.90%)	163,990 (67.90%)	11,555 (39.13%)	57,775 (39.13%)
Age at index date		Range: 10.01-24.99<br>1st-3rd quartile: 16.73 -21.87<br>Median: 19.25<br>Mean: 19.25<br>Standard deviation: 3.18	Range: 9.28-25.87<br>1st-3rd quartile: 16.71 - 21.88<br>Median: 19.26<br>Mean: 19.26<br>Standard deviation: 3.20	Range: 10.00-24.99<br>1st-3rd quartile: 15.97-21.94<br>Median: 19.14<br>Mean: 18.81<br>Standard deviation: 3.78	Range: 9.20-25.86<br>1st-3rd quartile: 15.96-21.94<br>Median: 19.14<br>Mean: 18.81<br>Standard deviation: 3.80
Age group at index date	10-12 years	176 (0.36%)	958 (0.40%)	1451 (4.91%)	7403 (5.01%)
	13-17 years	13,262 (27.46%)	65,909 (27.29%)	8047 (27.25%)	40,109 (27.16%)
	18-24 years	34,862 (72.18%)	174,633 (72.31%)	20,034 (67.84%)	100,148 (67.82%)
**Outcome event**					
Subsequent self-harm	1 month	3125 (6.47%)	33 (0.01%)	145 (0.49%)	17 (0.01%)
	3 months	4772 (9.88%)	109 (0.05%)	275 (0.93%)	46 (0.03%)
	1 year	8120 (16.81%)	464 (0.19%)	593 (2.01%)	195 (0.13%)
Suicide attempt	1 month	1928 (3.99%)	25 (0.01%)	93 (0.31%)	19 (0.01%)
	3 months	3207 (6.64%)	83 (0.03%)	190 (0.64%)	47 (0.03%)
	1 year	5996 (12.41%)	355 (0.15%)	451 (1.53%)	178 (0.12%)
Suicide	1 month	22 (0.05%)	0	≤5	0
	3 months	54 (0.11%)	0	8 (0.03%)	0
	1 year	145 (0.30%)	0	20 (0.07%)	0

Note: Individuals without a self-harm on the index date were matched with ratio 1 self-harm:5 no self-harm on sex and birth year. Matched unexposed were Swedish born, to ensure appropriate follow-up.

Table S4: Hazard ratios of subsequent self-harm, suicide attempt and suicide within the year following initial self-harm, comparing individuals with index self-harm defined based on intention vs matched without a self-harm

Outcome	Exposure group	Events	Total person years (1000)	Incidence rate per 1000 person years (95% confidence interval)[a]	Hazard ratio (95% confidence interval)[a]
Subsequent self-harm	No self-harm	195	147.23	1.32 (1.15, 1.52)	1 (ref)
	Index self-harm based on events of **undetermined intent**	593	29.04	20.42 (18.81, 22.13)	15.73* (13.35, 18.53)
	No self-harm	464	240.67	1.93 (1.76, 2.11)	1 (ref)
	Index self-harm based on events of **determined intent**	8120	42.18	192.53 (188.36, 196.76)	101.09* (91.44, 111.75)
Suicide attempt	No self-harm	178	147.22	1.21 (1.04, 1.40)	1 (ref)
	Index self-harm based on events of **undetermined intent**	451	29.14	15.47 (14.08, 16.97)	12.87* (10.81, 15.32)
	No self-harm	355	240.73	1.47 (1.33, 1.64)	1 (ref)
	Index self-harm based on events of **determined intent**	5996	43.90	136.58 (133.15, 140.08)	92.67* (82.86, 103.65)
Suicide	No self-harm	0	147.32	0	1 (ref)
	Index self-harm based on events of **undetermined intent**	20	29.43	0.68 (0.42, 1.05)	N/A
	No self-harm	0	240.91	0	1 (ref)
	Index self-harm based on events of **determined intent**	145	48.06	3.02 (2.55, 3.55)	N/A

Note: Individuals without a self-harm on the index date were matched with ratio 1 self-harm:5 no self-harm on sex, and birth year. Matched unexposed were Swedish born, to ensure appropriate follow-up.

[a] Incidence rates and hazard ratios were not estimated groups without any outcome events

*P-value <0.005, indicating hazard ratios statistically significantly different from 1

III. SUPPLEMENTARY FIGURES

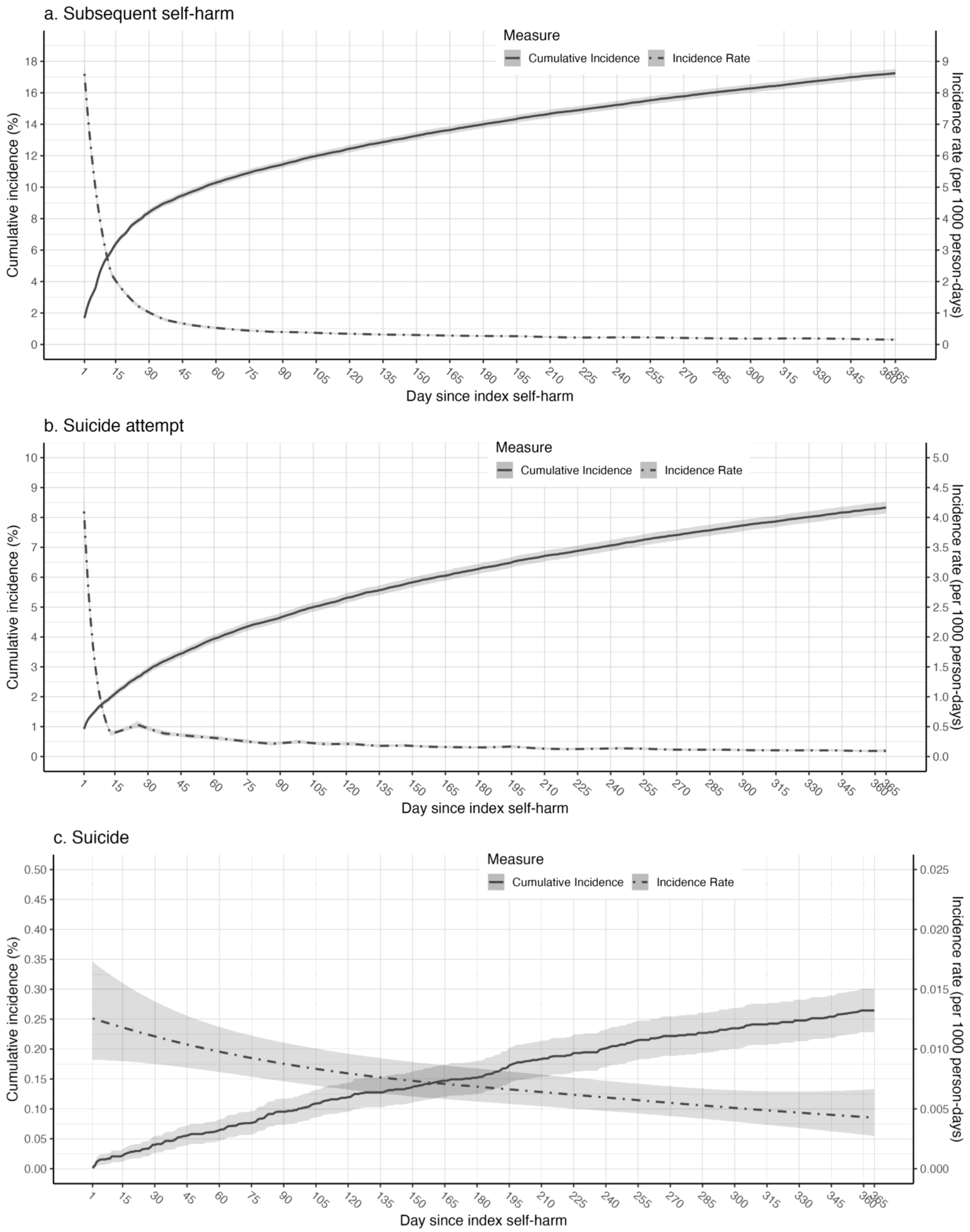

Figure S1: Incidence of outcomes across calendar day within 1 year since the index self-harm
Incidence of a) subsequent self-harm; b) Suicide attempt; c) Suicide
Cumulative incidence (solid lines, y axis on the left) and Incidence rate (dot-dashed line, y-axis on the right). Estimates from crude models not adjusted for any covariate. Shading regions show 95% confidence intervals of the estimates. Cumulative incidence was estimated using the Kaplan-Meier method; the line shows the proportion (%) of individuals with an outcome among individuals who were still at risk at corresponding day on the x-axis.

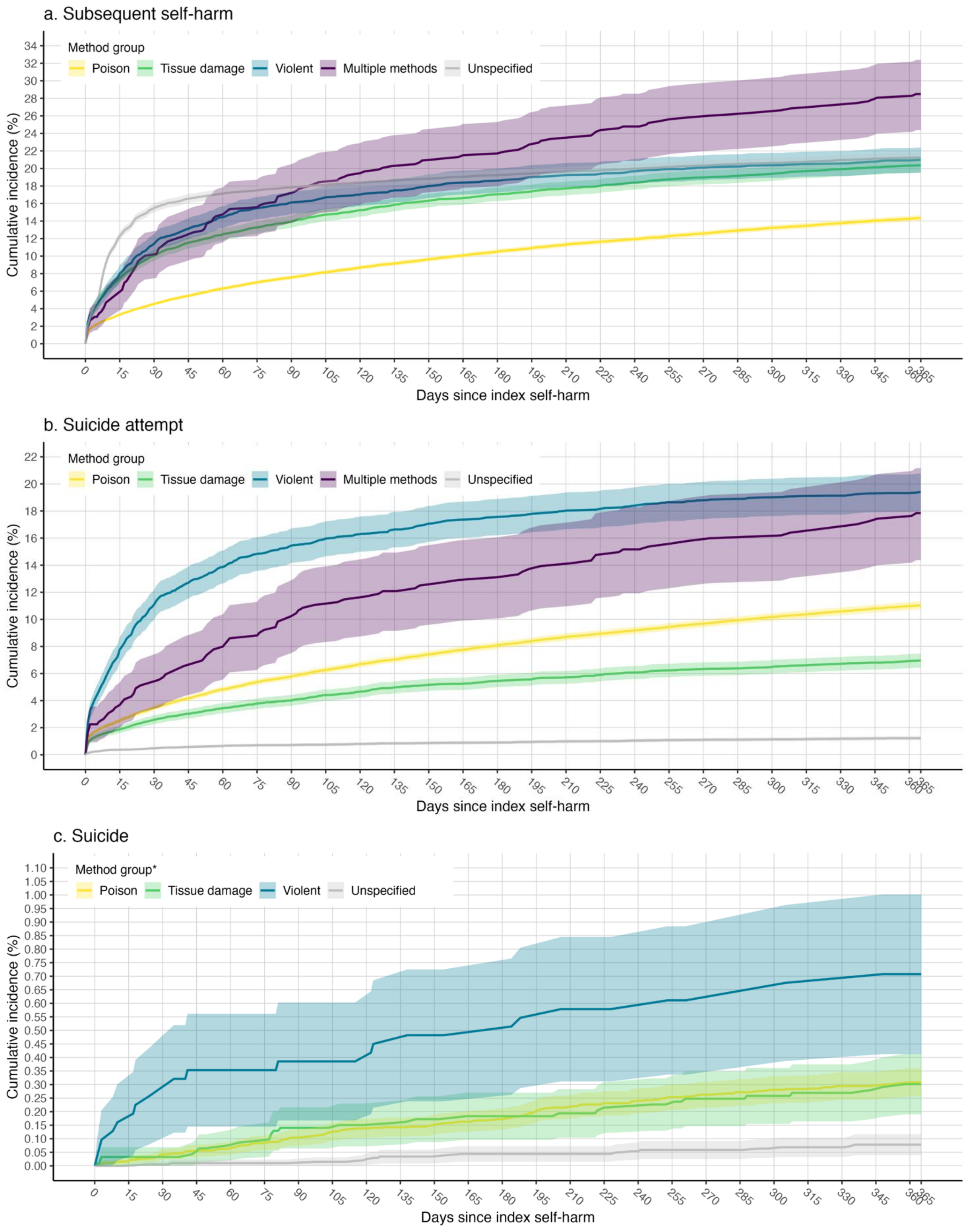

Figure S2: Cumulative incidence by method of self-harm over calendar day since the index self-harm
Incidence of a) subsequent self-harm; b) Suicide attempt; c) Suicide
Cumulative incidence was estimated from crude models not adjusted for any covariate, using the Kaplan-Meier method. Colours represent data separately by age groups. The lines show the proportion (%) of individuals who experienced the outcome among those still at risk on each corresponding day shown on the x-axis. Shading regions show 95% confidence intervals of the estimates.
*The 'Multiple method' group was not presented due to confidentiality concerns, as the number of cases was too low.

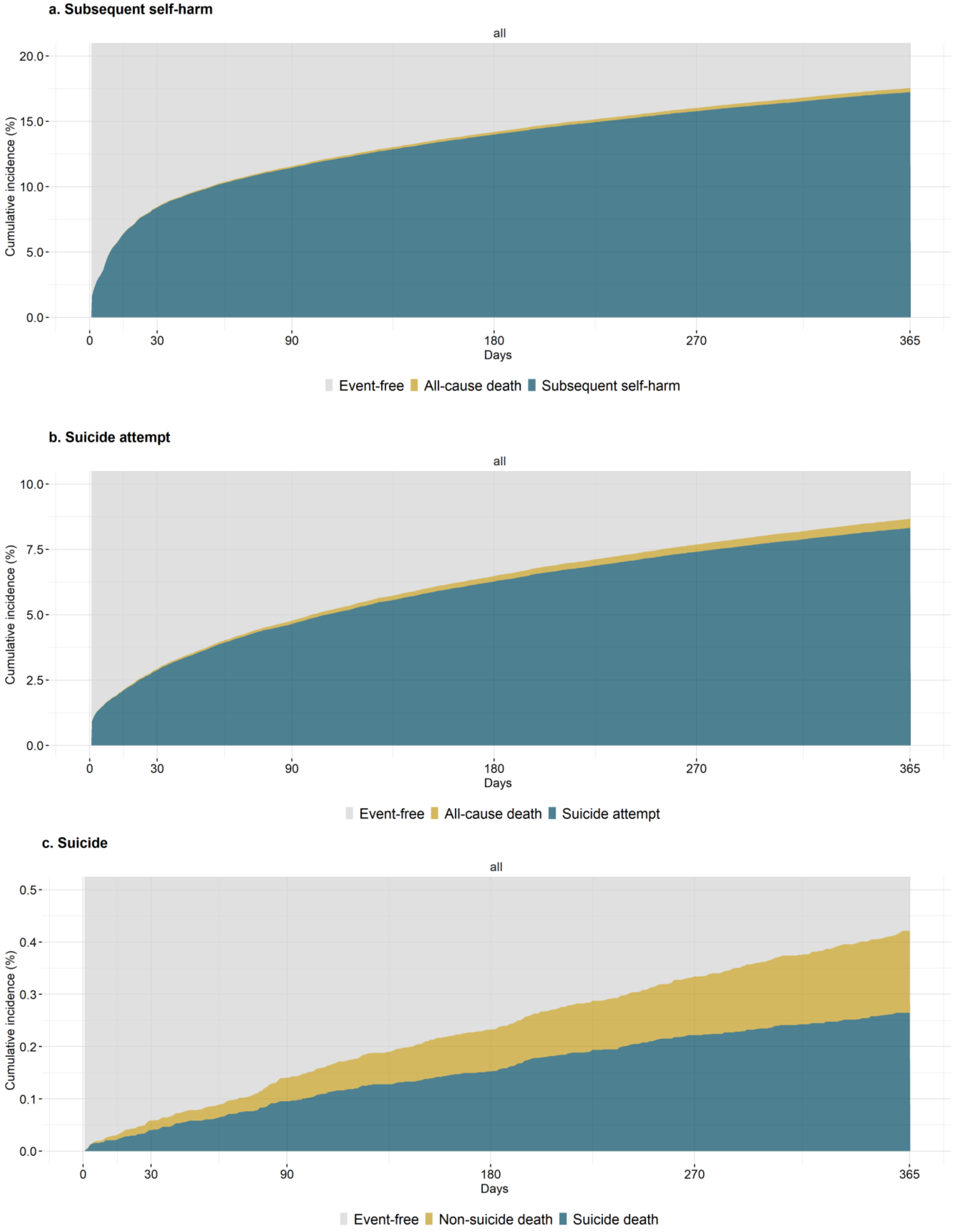

Figure S3: Cumulative incidence of outcomes over calendar day since the index self-harm considering competing events

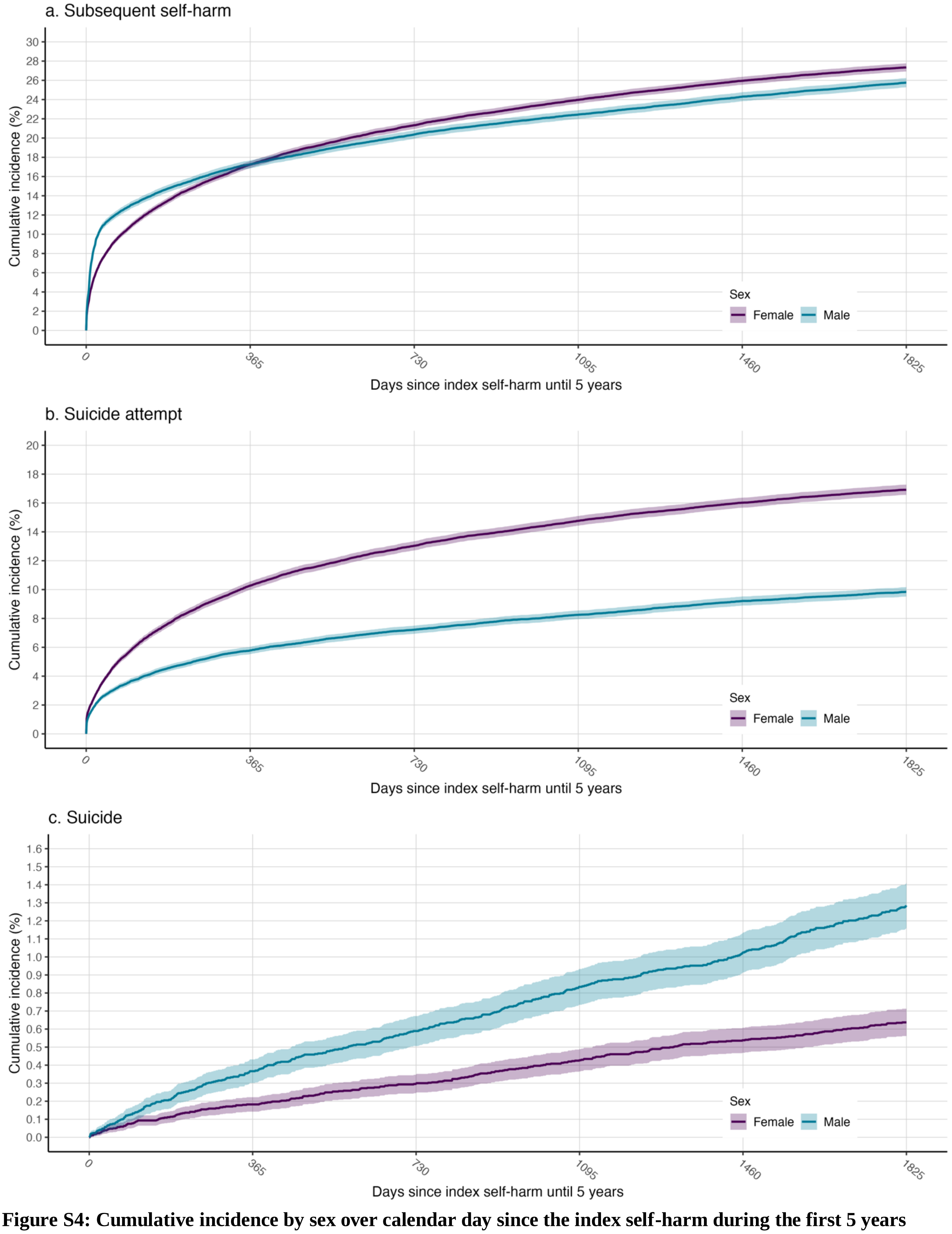

Figure S4: Cumulative incidence by sex over calendar day since the index self-harm during the first 5 years
Incidence of a) subsequent self-harm; b) suicide attempt; c) Suicide
Cumulative incidence was estimated from crude models not adjusted for any covariate, using the Kaplan-Meier method. Colours represent sex. The lines show the proportion (%) of individuals with an outcome among individuals who were still at risk at corresponding day on the x-axis. Shading regions show 95% confidence intervals of the estimates.

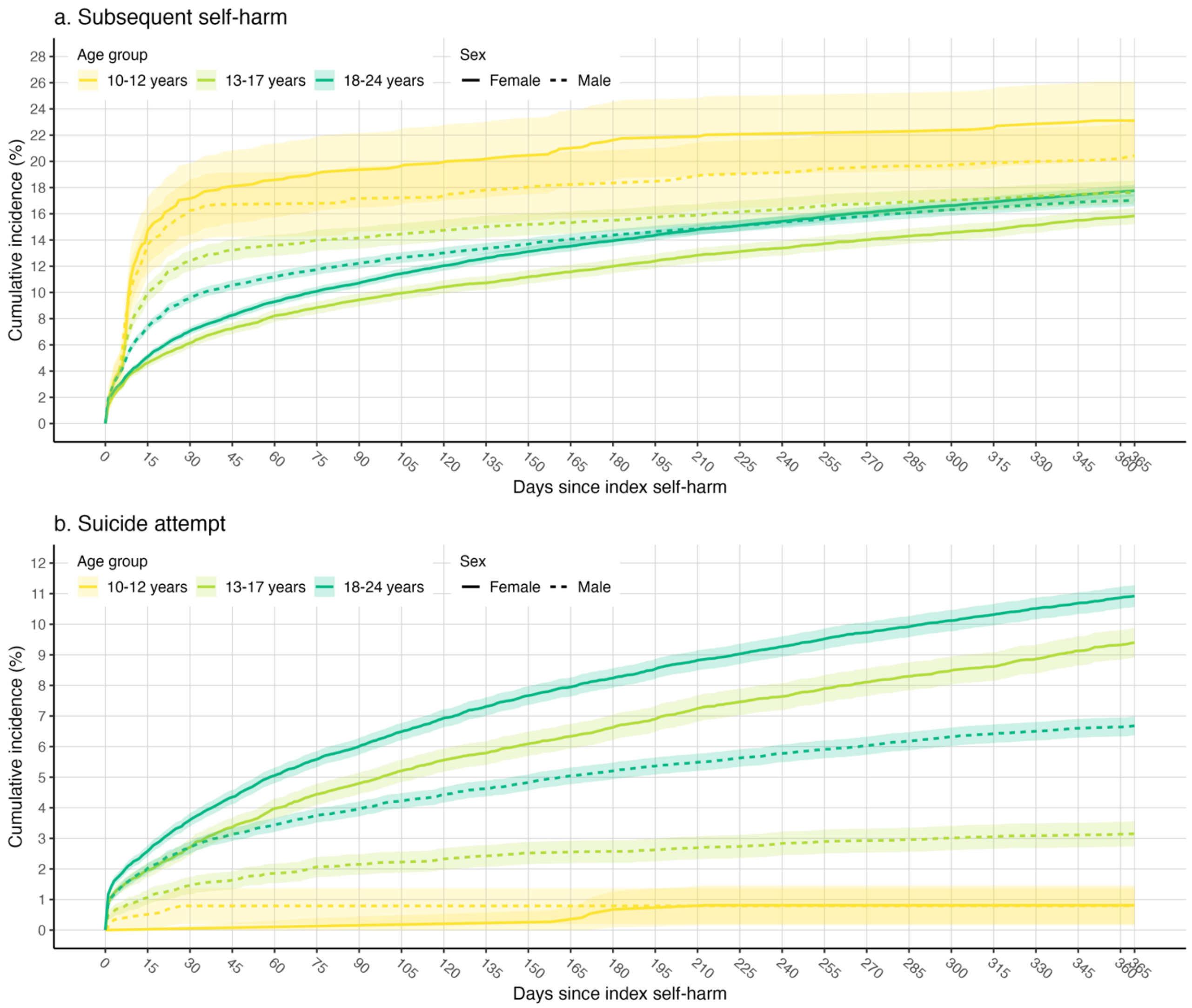

Figure S5: Cumulative incidence by sex and age group over calendar day since the index self-harm

Incidence of a) subsequent self-harm; b) Suicide attempt

Cumulative incidence was estimated from crude models not adjusted for any covariate, using the Kaplan-Meier method. Colours represent age groups; line types represent sex. The lines show the proportion (%) of individuals with an outcome among individuals who were still at risk at corresponding day on the x-axis. Shading regions show 95% confidence intervals of the estimates.

References

1. Katki HA, Berndt SI, Machiela MJ, Stewart DR, Garcia-Closas M, Kim J, et al. Increase in power by obtaining 10 or more controls per case when type-1 error is small in large-scale association studies. BMC Med Res Methodol. 2023 June 29;23(1):153.

2. Utility Functions, Datasets and Extended Examples for Survival Analysis.

3. Ludvigsson JF, Almqvist C, Bonamy AKE, Ljung R, Michaëlsson K, Neovius M, et al. Registers of the Swedish total population and their use in medical research. Eur J Epidemiol. 2016 Feb;31(2):125–36.

4. Ludvigsson JF, Andersson E, Ekbom A, Feychting M, Kim JL, Reuterwall C, et al. External review and validation of the Swedish national inpatient register. BMC Public Health. 2011 June 9;11:450.

5. Brooke HL, Talbäck M, Hörnblad J, Johansson LA, Ludvigsson JF, Druid H, et al. The Swedish cause of death register. Eur J Epidemiol. 2017 Sept;32(9):765–73.
